# Prognostic implications of blood lactate concentrations after cardiac arrest: a retrospective study

**DOI:** 10.1186/s13613-017-0321-2

**Published:** 2017-10-06

**Authors:** Antonio Maria Dell’Anna, Claudio Sandroni, Irene Lamanna, Ilaria Belloni, Katia Donadello, Jacques Creteur, Jean-Louis Vincent, Fabio Silvio Taccone

**Affiliations:** 1 Department of Intensive Care, Erasme Hospital, Université Libre de Bruxelles, Route de Lennik 808, 1070 Brussels, Belgium; 2 Department of Anesthesiology and Intensive Care, Catholic University School of Medicine, Largo Agostino Gemelli 8, 00168 Rome, Italy; 3 Anaesthesia and Intensive Care B, Department of Surgery, Dentistry, Paediatrics and Gynaecology, University of Verona, AOUI-University Hospital Integrated Trust of Verona, P.le L.A. Scuro 10, 37134 Verona, Italy

**Keywords:** Neurological outcome, Cardiopulmonary resuscitation, In-hospital cardiac arrest, Hyperlactataemia

## Abstract

**Background:**

Elevated lactate concentration has been associated with increased mortality after out-of-hospital cardiac arrest (CA). We investigated the variables associated with high blood lactate concentrations and explored the relationship between blood lactate and neurological outcome in this setting.

**Methods:**

This was a retrospective analysis of an institutional database that included all adult (> 18 years) patients admitted to a multidisciplinary Department of Intensive Care between January 2009 and January 2013 after resuscitation from CA. Blood lactate concentrations were collected at hospital admission and 6, 12, 24 and 48 h thereafter. Neurological outcome was evaluated 3 months post-CA using the Cerebral Performance Category (CPC) score: a CPC of 3–5 was used to define a poor outcome.

**Results:**

Of the 236 patients included, 162 (69%) had a poor outcome. On admission, median lactate concentrations (5.3[2.9–9.0] vs. 2.5[1.5–5.5], *p* < 0.001) and cardiovascular sequential organ failure assessment (cSOFA) score (3[0–4] vs. 0[0–3], *p* = 0.003) were higher in patients with poor than in those with favourable outcomes. Lactate concentrations were higher in patients with poor outcomes at all time points. Lactate concentrations were similar in patients with out-of-hospital and in-hospital CA at all time points. After adjustment, high admission lactate was independently associated with a poor neurological outcome (OR 1.18, 95% CI 1.08–1.30; *p* < 0.001). In multivariable analysis, use of vasopressors and high PaO_2_ on admission, longer time to return of spontaneous circulation and altered renal function were associated with high admission lactate concentrations.

**Conclusions:**

High lactate concentrations on admission were an independent predictor of poor neurological recovery post-CA, but the time course was not related to outcome. Prolonged resuscitation, use of vasopressors, high PaO_2_ and altered renal function were predictors of high lactate concentrations.

**Electronic supplementary material:**

The online version of this article (doi:10.1186/s13613-017-0321-2) contains supplementary material, which is available to authorized users.

## Background

Every year, between 37 and 100 per 100,000 inhabitants have an out-of-hospital cardiac arrest (OHCA) in Western countries, with approximately 10% surviving to hospital discharge [1, 2]. Despite the more immediate availability of advanced life support, survival to hospital discharge after in-hospital CA (IHCA) does not exceed 15–20% [3, 4]. High-quality cardiopulmonary resuscitation (CPR) and early defibrillation, when indicated, are essential to achieve a good outcome [5]. However, when return of spontaneous circulation (ROSC) is achieved, there is still uncertainty about which therapeutic interventions most effectively improve the survival and neurological recovery of these patients.

Blood lactate concentrations may represent a marker of cellular hypoxia after CA. Patients with high blood lactate concentrations on admission after CA are more likely to die than those with lower concentrations [6, 7]. In this setting, blood lactate concentrations may be considered as a marker of prolonged hypoperfusion or poor resuscitation. Other studies have also suggested that a faster decrease in lactate concentrations during the early hours after resuscitation may be correlated with better survival [8–10]. This decrease may reflect more rapid haemodynamic stabilization with therapy. Nevertheless, these studies included only patients with OHCA and the importance of lactate concentrations in IHCA survivors remains uncertain. As the underlying causes and management of OHCA and IHCA are different (e.g. cardiac vs. non-cardiac causes, different delays for interventions, different types of initial rhythm, presence of pre-existing medical conditions), the absolute initial lactate concentrations, as well as changes in lactate concentrations over time, may vary accordingly and potentially have different prognostic value. Few data are available on the impact of lactate concentrations and their evolution on neurological recovery, and the variables associated with high blood lactate after CA have not been well studied [7].

The aims of this study were, therefore, to: (a) evaluate whether any lactate-related variable (i.e. admission value, peak lactate, decreased concentration over time, area under the curve of concentrations over 48 h) was associated with neurological outcome; (b) identify the variables associated with high lactate concentrations after CA; and (c) explore the differences in lactate concentrations between IHCA and OHCA.

## Methods

### Study population

We retrospectively reviewed prospectively collected data from all adult (> 18 years of age) patients who were admitted to the 35-bed medical/surgical Department of Intensive Care of Erasme Hospital after OHCA or IHCA (excluding patients with CA occurring in the ICU) between January 2009 and January 2013. The local Institutional Review Board approved the study and waived the need for informed consent.

### Routine post-resuscitation care

A standardized institutional protocol is used in all post-cardiac arrest patients (1). All comatose patients, irrespective of CA location and initial rhythm, are treated with targeted temperature management (TTM) for 24 h, with a target temperature of 32–34 °C. Cooling is started immediately after ICU admission with a combination of cold fluid bolus (20–30 ml/kg of a crystalloid solution in 30 min) and a circulating water blanket device (Medi-Therm II, Gaymar, USA). Body temperature is measured using invasive haemodynamic monitoring (PiCCO, Pulsion, Munich, Germany) when in place (need determined by attending physician depending on patient condition) or a rectal temperature probe. Analgo-sedation is provided using midazolam (0.03–0.1 mg/kg/h) and morphine (0.1–0.3 mg/kg/h) infusions. Cisatracurium is administered to control shivering in the induction phase (as a bolus of 0.15 mg/kg) and, if needed, as a continuous infusion thereafter (1–3 mcg/kg/min). After 24 h of cooling, rewarming (0.3–0.5 °C/h) is performed passively and sedation discontinued when body temperature reaches 37 °C.

Patients are kept in a 30° semi-recumbent position; ventilation is set to keep PaCO_2_ between 35 and 45 mmHg and SpO_2_ > 94%. Transesophageal echocardiography is routinely performed within the first 6–18 h after ICU admission. Blood glucose is kept between 110 and 150 mg/dL and mean arterial pressure maintained > 65–70 mm Hg using fluids, dobutamine and/or noradrenaline. Intra-aortic balloon counterpulsation (IABP) or extracorporeal membrane oxygenation (ECMO) is used in cases of severe cardiogenic shock.

### Neurological assessment and withdrawal of life support

After rewarming and discontinuation of sedation, repeated neurological examination and standard or continuous electroencephalography (EEG) together with somatosensory evoked potentials (SSEPs) at day 3 are performed (“multimodal” approach). After the first 72 h, a decision to withdraw life support may be taken in comatose patients based on: (a) bilateral absence of the N20 wave of SSEPs; (b) presence of status myoclonus (defined as continuous and generalized myoclonus persisting for ≥ 30 min [11]) in combination with EEG abnormalities (generalized discharges or burst suppression); (c) refractory (i.e. resistant to two antiepileptic drugs and continuous intravenous sedative administration) status epilepticus occurring from a flat or burst suppression background. The medical charts of patients were reviewed to classify deaths as “non-neurological” (i.e. persistent shock or multiple organ failure in the absence of neurological injury or before neurological prognostication could be assessed) or as neurological (i.e. severe anoxic brain injury) [12].

### Data collection

We collected patient demographics, comorbidities and Utstein variables, including CA location, initial rhythm, bystander CPR and time to ROSC. After hospital admission, blood lactate concentrations are routinely measured with arterial blood gases, glucose and electrolytes every 3–4 h by point-of-care analysers (GEM Premier 4000; Instrumentation Laboratory, Bedford, MA, USA). We recorded the initial lactate concentration, measured within 1 h after hospital admission, and values measured at 6, 12, 24 and 48 h after admission, with a margin of ± 1 h. At the same time points, we also recorded blood gases, heart rate (HR) and MAP and calculated a sequential organ failure assessment (SOFA) score [13] from the ICU patient data monitoring system (Picis Critical Care Manager, Picis Inc., Wakefield, USA). The SOFA score was calculated excluding the neurological component because of the confounding effect of sedation. Shock was defined as a cardiovascular SOFA (cSOFA) of 3 or 4.

All available lactate measurements during the first 48 h or until death (if the patient died within 48 h) were plotted to calculate the area under the curve (AUC_0-48_). We report the highest value recorded during the first 48 h as the “peak lactate”. The absolute decrease in lactate concentrations from admission was calculated at 6, 12 and 24 h, using the following formulas: [(lactate at 6 h—lactate on admission)/6] mEql/L, [(lactate at 12 h—lactate on admission)/12] mEq/L and [(lactate at 24 h—lactate on admission)/24] mEq/L. Similarly, the “relative” decrease in lactate concentrations was calculated as follows: [(lactate at 6 h—lactate on admission)/lactate on admission × 100]%, [(lactate at 12 h—lactate on admission)/lactate on admission × 100]% and [(lactate at 24 h—lactate on admission)/lactate on admission × 100]% [8, 14].

### Outcome assessment

Outcome was assessed by the neurological outcome at 3 months, determined using the Cerebral Performance Category score (CPC; 1 = no neurological disability, 2 = mild neurological disability, 3 = severe neurological impairment, 4 = vegetative state, 5 = death) [15]. The CPC was prospectively evaluated during follow-up visits or by telephone interview with the general practitioner. A favourable outcome was defined as a CPC 1 or 2 and poor outcome as CPC 3–5.

### Statistical analysis

Statistical analyses were performed using IBM SPSS Statistics 21 for Macintosh. Descriptive statistics were computed for all study variables. A Kolmogorov–Smirnov test was used, and histograms and normal quantile plots were examined to verify the normality of distribution of continuous variables. Data are presented as count (percentage), mean (± standard deviation) or median [25th–75th percentiles], as appropriate. Differences between groups were assessed using a Chi-square or Fisher’s exact test for categorical variables, as appropriate, and a *T* test or a Wilcoxon rank test for continuous variables. Data from repeated measures (lactate, HR, MAP, cSOFA on admission and at 6, 12, 24 and 48 h, thereafter) were analysed using a two-way ANOVA, or the Friedman ANOVA for data that were not normally distributed. Differences between groups for each time point were explored using a Mann–Whitney *U* test for nonparametric data. Association between continuous variables was evaluated using linear regression. Multivariable logistic regression was performed to identify factors independently associated with high blood lactate concentrations on admission. Multivariate binomial backward logistic regression was used to assess whether blood lactate on admission or decrease in lactate concentrations was independently correlated with favourable outcome. The discriminative ability of variables identified to predict neurological outcome by multivariate analysis was evaluated using receiver operating characteristic (ROC) curves with the corresponding AUC. Subgroup analyses included comparisons between IHCA and OHCA, patients with shockable and non-shockable rhythm and patients with shock (defined as cSOFA ≥ 3) and without shock on admission. A *p* < 0.05 was considered as statistically significant.

## Results

### General characteristics of the study cohort

Among 244 consecutive patients admitted to our ICU after CA, 236 were included in the study (Additional file 1: Figure S1). The mean age of our cohort was 63 years; 155 (66%) patients were male and 137 (58%) had an OHCA; 162 (69%) patients had a poor neurological outcome at 3 months (Table 1).

**Table 1:** Characteristics of study population according to neurological outcome at 3 months

Parameter	All patients (236)	CPC 1–2 (74)	CPC 3–5 (162)	*p* value
*General*				
Age (years)	63 ± 15	59 ± 14	64 ± 16	0.03
Male, *n* (%)	155 (60%)	57 (77%)	98 (61%)	0.02
*Comorbidities*				
COPD/asthma, *n* (%)	43 (18%)	15 (20%)	28 (17%)	0.59
Heart disease, *n* (%)	107 (46%)	40 (54%)	67 (42%)	0.09
Diabetes, *n* (%)	50 (21%)	21 (28%)	29 (18%)	0.08
Chronic renal failure, *n* (%)	38 (16%)	12 (16%)	26 (16%)	1
Liver cirrhosis, *n* (%)	14 (6%)	6 (8%)	8 (65%)	0.38
Immunosuppression, *n* (%)	28 (12%)	5 (7%)	23 (14%)	0.13
*Cardiac arrest*				
OHCA, *n* (%)	137 (58%)	43 (58%)	94 (58%)	1
VF/VT, *n* (%)	100 (42%)	57 (77%)	43 (27%)	<0.001
Bystander CPR, *n* (%)	136 (58%)	54 (73%)	82 (52%)	0.03
Time to ROSC (min)	19 ± 14	17 ± 13	20 ± 14	0.10
*ABG*				
Lactate admission (mEq/L)	4.3 [2–8.5]	2.5 [1.5–5.5]	5.3 [2.9–9.0]	<0.001
Lactate 6 h (mEq/L)	2.7 [1.6–5]	2.0 [1.3–2.9]	3.3 [1.8–5.9]	<0.001
Lactate 12 h (mEq/L)	2.2 [1.4–4.2]	1.6 [1.2–2.3]	2.8 [1.6–5.3]	<0.001
Lactate 24 h (mEq/L)	1.5 [1.1–2.8]	1.3 [1.0–1.7]	1.8 [1.3–3.2]	<0.001
Lactate 48 h (mEq/L)	1.4 [1.0–2.0]	1.1 [0.8–1.7]	1.6 [1.1–2.5]	0.001
aDLC_0-6_ (mEq/L*h)	0.2 [0–0.6]	0.1 [0–0.5]	0.2 [0–0.6]	0.16
aDLC_0-12_ (mEq/L*h)	0.1 [0–0.3]	0.1 [0–0.2]	0.2 [0–0.4]	0.006
aDLC_0-24_ (mEq/L*h)	0.1 [0–0.3]	0.1 [0–0.1]	0.1 [0–0.3]	0.008
rDLC_0-6_ (%)	25 [−5 to 54]	24 [−8 to 56]	24 [−5 to 53]	0.98
rDLC_0-12_ (%)	37 [2–62]	28 [−7 to 59]	40 [33–47]	0.08
rDLC_0-24_ (%)	53 [18–71]	48 [25–68]	55 [17–77]	0.64
AUC_0-48_, mEq/L*h	4.7 ± 4.2	3.74 ± 2.53	5.17 ± 4.72	0.016
Peak lactate, mEq/L	5 [3–9]	3.6 [2.2–6.3]	6.7 [3.8–10.2]	<0.001
pH on admission	7.25 [7.16–7.36]	7.27 [7.18–7.37]	7.25 [7.15–7.34]	0.09
PaO_2_ on admission (mmHg)	121 [77–228]	143 [84–220]	118 [77–232]	0.54
PaCO_2_ on admission (mmHg)	42 ± 14	43 ± 12	42 ± 15	0.41
*Haemodynamics*				
HR on admission (bpm)	89 ± 21	89 ± 19	88 ± 22	0.75
MAP on admission (mmHg)	90 ± 24	98 ± 23	88 ± 24	0.004
Vasopressors/inotropes on admission	130 (55%)	30 (40%)	100 (62%)	0.003
Vasopressors/inotropes at 6 h	139 (59%)	32 (43%)	107 (66%)	0.01
Vasopressors/inotropes at 12 h	150 (64%)	37 (50%)	113 (70%)	0.003
Vasopressors/inotropes at 24 h	129 (60%)	37 (50%)	92 (66%)	0.03
Vasopressors/inotropes at 48 h	131 (61%)	32 (43%)	99 (71%)	<0.001
*SOFA scores*				
SOFA score on admission	4 [2–7]	4 [2–5]	4 [2–8]	0.08
cSOFA admission	2 [0–4]	0 [0–3]	3 [0–4]	0.003
hSOFA admission	0 [0–0]	0 [0–0]	0 [0–1]	0.001
hSOFA maximum	0 [0–0]	0 [0–0]	0 [0–1]	0.001
*Interventions*				
IABP, *n* (%)	18 (8%)	9 (12%)	9 (5.6%)	0.19
ECMO, *n* (%)	15 (6%)	2 (3%)	13 (8%)	0.15
CRRT, *n* (%)	32 (14%)	3 (4%)	29 (18%)	0.004
*Outcomes*				
ICU LOS (days)	3 [1–6]	5 [3–11]	2 [1–4]	<0.001
Neurological cause of death	87 (36%)	–	87 (54%)	<0.001

*CPC* Cerebral Performance Category, *CPC 1–2* favourable outcome, *CPC 3–5* poor outcome, *MAP* mean arterial pressure, *SOFA* sequential organ failure assessment (without the neurological subscore), *cSOFA* cardiovascular SOFA score, *ICU* intensive care unit, *LOS* length of stay, *HR* heart rate, *ABG* arterial blood gas analysis, *aDLC* absolute decrease in lactate concentration over time expressed as mEq/L*h, *rDLC* relative decrease in lactate concentration over time expressed as %, *AUC*_*0-48*_ area under the curve of lactate concentrations for the first 48 h after admission, *COPD* chronic obstructive pulmonary disease, *IABP* intra-aortic balloon counterpulsation, *ECMO* extracorporeal membrane oxygenation, *CRRT* continuous renal replacement therapy, *hSOFA* hepatic SOFA subscore, *VF/VT* ventricular fibrillation/ventricular tachycardia, *OHCA* out-of-hospital cardiac arrest, *CPR* cardiopulmonary resuscitation, *ROSC* return of spontaneous circulation

### Blood lactate concentrations and outcome

The median blood lactate concentration on admission was 4.3[2.0–8.5] mEq/L. Lactate concentrations were higher on admission (5.3[2.9–9.0] vs. 2.5[1.5–5.5], *p* < 0.001) and at each time point thereafter in patients with poor than in those with favourable neurological outcome (Table 1 and Additional file 1: Figure S2). Peak lactate concentration (6.7[3.8–10.2] vs. 3.6[2.2–6.3], *p* < 0.001) and lactate AUC_0-48_ (3.9[2.4–6] vs. 3.2[2.4–4.5] mEq/L*h, *p* = 0.027) were also higher in patients with poor than in those with favourable neurological outcome. There were no differences in the relative changes in blood lactate concentrations at any time point between patients with poor and those with favourable neurological outcomes (Table 1).

Blood lactate levels on admission were somewhat higher in patients who died from a non-neurological cause (*n* = 72) than in those who died of neurological causes (*n* = 87), but the differences did not reach statistical significance (6.5 [3.2–9.1 vs. 4.8 [2.4–9.4] mEq/L; *p* = 0.16). Changes in blood lactate concentrations from admission to 12 h were similar in these two groups (data not shown).

In multivariable analysis, high admission blood lactate concentration was associated with significantly higher odds of poor neurological outcome (OR 1.18[1.08–1.30], *p* < 0.001) (Table 2). Using an ROC curve, blood lactate on admission had a predictive accuracy of 0.69 (95% CI 0.62–0.75) for poor neurological outcome (Additional file 1: Figure S3).

**Table 2:** Logistic regression analysis to identify predictors of poor neurological outcome (CPC 3–5) at 3 months

Parameter	OR	95% CI	*p* value
Bystander CPR	0.30	0.14–0.64	0.002
Shockable rhythm	0.11	0.05–0.23	<0.001
Lactate on admission, mEq/L	1.18	1.08–1.30	<0.001

*OR* odds ratio, *CI* confidence interval, *CPR* cardiopulmonary resuscitation

### Subgroup analyses

Lactate concentrations were similar in IHCA and OHCA patients on admission and at subsequent time points (Table 3). In patients with IHCA, but not in those with OHCA, blood lactate concentrations were higher in patients with a poor neurological outcome than in those with a favourable outcome at all time points (Additional file 1: Table S1). Lactate concentrations on admission and at subsequent time points were similar in patients with shockable than those with non-shockable rhythms (Additional file 1: Table S2). Blood lactate concentrations were higher on admission and throughout the study period in patients with shock on admission than in those without (Additional file 1: Table S3).

**Table 3:** Characteristics of patients according to arrest location (out of hospital [OHCA] vs. in hospital [IHCA])

Parameter	OHCA (137)	IHCA (99)	P value
*General*			
Age	61 ± 15	63 ± 15	040
Male	92 (67%)	64 (64%)	0.78
*Comorbidities*			
COPD/asthma, *n* (%)	24 (17%)	19 (19%)	0.74
Heart disease, *n* (%)	62 (45%)	45 (45%)	1.00
Diabetes, *n* (%)	31 (23%)	19 (19%)	0.63
Chronic renal failure, *n* (%)	21 (15%)	17 (17%)	0.51
Liver cirrhosis, *n* (%)	6 (4%)	8 (8%)	0.27
Immunosuppression, *n* (%)	17 (12%)	11 (11%)	0.84
*Cardiac arrest*			
VF/VT	65 (47%)	35 (35%)	0.086
Bystander CPR	57 (42%)	79 (80%)	<0.001
Time to ROSC (min)	21.4 ± 12.8	16.7 ± 14.3	0.011
*ABG*			
Lactate admission (mEq/L)	4.3 [2.2–7.9]	4.8 [1.9–8.6]	0.81
Lactate 6 h (mEq/L)	2.7 [1.7–4.7]	2.7 [1.6–5.5]	0.51
Lactate 12 h (mEq/L)	2.2 [1.5–4.2]	2.3 [1.4–4.1]	0.78
Lactate 24 h (mEq/L)	1.5 [1.1–3.0]	1.4 [1.0–2.5]	0.605
Lactate 48 h (mEq/L)	1.4 [1.0–2.0]	1.4 [1.0–2.3]	0.415
aDLC_0-6_ (mEq/L*h)	.150 [−.03 to .583]	.167 [0–.517]	0.88
aDLC_0-12_ (mEq/L*h)	0.14 [0–0.3]	0.12 [0–0.3]	0.55
aDLC_0-24_ (mEq/L*h)	.075 [.004–.192]	.073 [.018–.228]	0.469
rDLC_0-6_ (%)	28 [−9 to 55]	22 [0–50]	0.75
rDLC_0-12_ (%)	38 [0–64]	33 [7–61]	0.65
rDLC_0-24_ (%)	51.6 [10.5–75.3]	56 [25.5–70.5]	0.558
AUC_0-48_, mEq/L*h	4.6 ± 4	4.7 ± 4.4	0.901
Peak lactate, mEq/L	6.4 ± 3.8	6.9 ± 5.2	0.001
pH admission	7.25 [7.16–7.34]	7.27 [7.19–7.40]	0.038
PaO_2_ admission (mmHg)	124 [82–228]	116 [70–234]	0.289
PaCO_2_ admission (mmHg)	43.5 ± 14.8	40.4 ± 12.7	0.101
*Haemodynamics*			
HR admission	87 ± 22	91 ± 19	0.120
MAP admission (mmHg)	96 ± 25	84 ± 20	<0.001
Vasopressors/inotropes admission	60 (44%)	70 (71%)	<0.001
Vasopressors/inotropes 6 h	67 (49%)	72 (73%)	<0.001
Vasopressors/inotropes 12 h	75 (55%)	75 (75%)	0.001
Vasopressors/inotropes 24 h	66 (52%)	63 (73%)	0.002
Vasopressors/inotropes 48 h	68 (53%)	63 (73%)	0.004
*SOFA score*			
SOFA admission	3.5 [2–5]	5 [3–8]	<0.001
cSOFA admission	0 [0–4]	3 [0–4]	<0.001
hSOFA baseline	0 [0–0]	0 [0–1]	<0.001
hSOFA maximum	0 [0–0]	0 [0–1]	0.002
*Interventions*			
IABP, *n* (%)	9 (7%)	9 (9%)	0.47
ECMO, *n* (%)	7 (5%)	8 (8%)	0.42
CRRT, *n* (%)	19 (14%)	13 (13%)	1
*Outcomes*			
ICU LOS (days)	2 [1–6]	4 [1–7]	0.23
Neurological cause of death, *n* (%)	61 (44%)	26 (26%)	0.008
Poor outcome at 3 months	94 (69%)	68 (69%)	0.711

*MAP* mean arterial pressure, *SOFA* sequential organ failure assessment (without the neurological subscore), *cSOFA* cardiovascular SOFA score, *ICU* intensive care unit, *LOS* length of stay, *HR* heart rate, *aDLC* absolute decrease in lactate concentration over time expressed as mEq/L*h, *rDLC* relative decrease in lactate concentration over time expressed as %; *AUC*_*0-48*_ area under the curve of lactate concentrations for the first 48 h since admission, *COPD* chronic obstructive pulmonary disease, *IABP* intra-aortic balloon counterpulsation, *ECMO* extracorporeal membrane oxygenation, *CRRT* continuous renal replacement therapy, *hSOFA* hepatic SOFA subscore, *VF/VT* ventricular fibrillation/ventricular tachycardia, *OHCA* out-of-hospital cardiac arrest, *CPR* cardiopulmonary resuscitation, *ROSC* return of spontaneous circulation

### Predictors of lactate concentrations and correlation with other haemodynamic variables

**Fig. 1:**
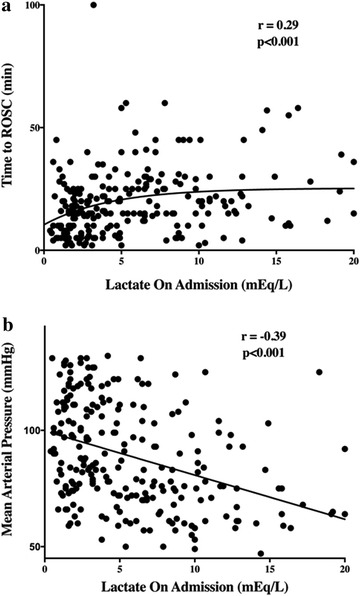
Panel A: Correlation between blood lactate concentration on admission and the time to return of spontaneous circulation (ROSC) (*r* = 0.29; *p* < 0.001). Panel B: Correlation between blood lactate concentration on admission and the first recorded mean arterial pressure (MAP) (*r* = −0.39; *p* < 0.001)

There was a weak correlation between blood lactate concentrations on admission and time to ROSC (*r* = 0.29, *p* < 0.001) and MAP on admission (*r* = −0.39, *p* < 0.001, Fig. 1). There was also a significant correlation between initial blood lactate concentration and absolute decrease in lactate concentrations at 6 and 24 h (Additional file 1: Figure S4). In a linear regression model, the use of vasopressors on admission, time to ROSC, initial PaO_2_ and renal SOFA on admission were independently associated with high admission lactate levels (Table 4); however, the model explained only 32% of the variance.

**Table 4:** Linear regression analysis for admission blood lactate

Parameter	Coefficient	*p* value
Renal SOFA	0.94 ± 0.23	<0.001
PaO_2_ on admission	0.006 ± 0.002	0.001
Time to ROSC (min)	0.067 ± 0.021	0.001
Vasopressors on admission	1.78 ± 0.62	0.004

*SOFA* sequential organ failure assessment, *ROSC* return of spontaneous circulation

## Discussion

Patients with a poor neurological outcome after CA had higher lactate values on admission and over the first 2 days than patients with a favourable outcome. Blood lactate concentration on admission remained a significant predictor of poor outcome even after adjustment for major confounders. Nonetheless, the relative decrease in lactate at 6, 12 and 24 h was not related to neurological outcome. In a linear regression model, use of vasopressors, PaO_2_, time to ROSC and renal SOFA were independently associated with high admission blood lactate concentration.

The pathophysiology of hyperlactataemia is complex and can involve tissue hypoperfusion, adrenergic hyperstimulation [16] and altered lactate clearance by the liver [17]. Interestingly, high lactate concentrations in our study were associated with renal dysfunction, which was probably a marker of organ failure [18] more than the kidney’s inability to clear lactate [16]. There was no association between lactate levels and the intensity of therapy (i.e. ECMO or IABP), although the number of treated patients was relatively limited. There was also no association of lactate on admission and hepatic dysfunction as assessed by the hepatic SOFA subscore. Moreover, increased PaO_2_ values were predictive of high lactate concentration on admission. Recent studies have suggested that very high PaO_2_ values (> 300 mmHg) may aggravate post-anoxic brain injury [19]. However, a specific threshold for oxygen toxicity has not yet been identified in clinical practice and the mechanisms relating oxygen toxicity to lactate production need to be further evaluated in this setting.

A high blood lactate concentration on admission was significantly correlated with poor neurological outcome. Similarly, in a large cohort of 443 patients, Lee et al. reported that a high blood lactate measured within 1 h of ROSC was correlated with CPC 3–5 at hospital discharge [7]. In 394 CA survivors enrolled over a 10-year period, Kliegel et al. [20] found a significant correlation between lactate at baseline, 24 and 48 h and neurological outcome. Moreover, blood lactate concentrations have been included, along with initial rhythm, estimated no-flow and low-flow intervals and creatinine levels, in a hospital admission predictive score for good neurological recovery after successful resuscitation from OHCA [21].

A decrease in lactate concentrations over time is a reliable marker of effective treatment in critically ill patients with shock [22–25]. As expected, we found that the higher the lactate on admission, the greater the decrease over time [26]. However, there was no relationship between lactate decrease and neurological outcome. Our findings contrast with those of Donnino et al. [14] who reported that lactate decrease at 6 and 12 h was more pronounced in survivors than in non-survivors, but no data on neurological recovery were available. Two studies have reported that the decrease in lactate at 12 h was an independent marker of good neurological outcome [8, 9]. The differences between these studies and our results may be explained by a lower median lactate on admission in our study (4.3 mEq/L in our study vs. 6 to 15 mEq/L in [8, 9, 14]), which is probably explained by a shorter duration of CA in our cohort of patients.

Of note, we also reported data in patients with IHCA, whereas most previous studies included only OHCA patients. Only Karagiannis et al. [27], in a small group of 28 patients after IHCA, described higher lactate concentrations at 6, 18 and 24 h after ROSC in non-survivors than in survivors, and a significantly lower percentage decrease in lactate at 6 and 12 h in non-survivors. In our cohort, lactate levels were similar in IHCA and OHCA patients at all time points. This is of interest because IHCA is usually secondary to different aetiologies than OHCA and associated with severe pre-existing comorbidities, which may alter the generalizability of outcome predictors for OHCA to the IHCA setting. Blood lactate values were also similar regardless of the initial rhythm, despite the fact that patients with non-shockable rhythms are more likely to have prior hypoxia or hypotension and, in general, a longer time from arrest to CPR [28, 29]. This suggests that the prognostic value of lactate levels on admission is independent of these CA characteristics and applicable to a wide CA population.

Our study has some limitations. First, given the retrospective design, we could not reliably account for some variables that may have influenced lactate concentrations, especially on admission, particularly CPR quality, total doses of adrenaline during CPR and fluid administration or fluid balance. However, consistent management in a single centre can reasonably exclude important differences in treatment strategies among patients. Moreover, we provided time to ROSC, but could not differentiate between “no-flow” and “low-flow” times, which could further influence the initial lactate levels (e.g. prolonged “no-flow” times should be associated with higher lactate values). Second, we evaluated a limited cohort of patients, including a heterogeneous population of patients with IHCA and OHCA, which may limit the generalizability of our findings. Third, our model could account for only one-third of the variability in lactate concentrations on admission, indicating that other factors that we did not account for—e.g. no-flow period, total dose of adrenaline given during CPR, vasopressors given during the ICU stay—may be involved in the pathophysiology of high lactate concentrations in this setting. Fourth, baseline lactate concentration may be related to the quality of resuscitation during CA, whereas lactate concentration at 12 or 24 h may be related more to the quality of critical care provided. Although this statement sounds logical, we have almost no data in the literature to confirm this hypothesis and the retrospective nature of our study precludes any additional analysis. Furthermore, the interpretation of baseline lactate could also be confounded by underlying conditions (e.g. sepsis) and/or aetiology of arrest (e.g. prolonged hypoxaemia before arrest). Fifth, lactate concentrations were available to the treating clinicians; although lactate concentrations are not used as a marker of poor prognosis in this setting, we cannot exclude that persistently high lactate concentrations may have encouraged limitation of invasive therapies in some of these patients, thus raising the risk of “self-fulfilling prophecy”. Finally, we evaluated liver function using the hepatic SOFA subscore, which is based on total bilirubin levels. Hypoxic hepatitis, as observed after CA, is, however, defined as an elevation of aminotransferases; nevertheless, the occurrence of hypoxic hepatitis is rare [30] and generally occurs 1–2 days after CA, which would be of limited interest for interpretation of admission lactate levels.

## Conclusions

Blood lactate concentrations in patients after CA were significantly higher in those with worse short- and long-term outcomes. However, decrease in lactate over time was not correlated with outcome. Interpretation of high lactate concentrations in CA survivors is complex and multifactorial.

## Additional file

**Additional file 1.** The additional file contains the supplementary figures and tables referred to in the text.

## Abbreviations

CA: cardiac arrest
CPR: cardiopulmonary resuscitation
EEG: electroencephalogram
HR: heart rate
IHCA: in-hospital cardiac arrest
MAP: mean arterial pressure
OHCA: out-of-hospital cardiac arrest
ROSC: return of spontaneous circulation
SOFA: sequential organ failure assessment
SSEP: somatosensory evoked potential
TTM: therapeutic temperature management
VF/VT: ventricular fibrillation/ventricular tachycardia
