# Supplementary material for: Prognostic implications of blood lactate concentrations after cardiac arrest: a retrospective study
Source: Ann Intensive Care. 2017 Oct 6;7:101. doi: 10.1186/s13613-017-0321-2 (PMC5630540; doi:10.1186/s13613-017-0321-2)

***Supplemental Digital Content***

**Prognostic Implications of Blood Lactate Concentrations After Cardiac Arrest: A Retrospective Study**

*Antonio Maria Dell’Anna, Claudio Sandroni, Irene Lamanna, Ilaria Belloni, Katia Donadello, Jacques Creteur, Jean-Louis Vincent, Fabio Silvio Taccone*

**Figure S1.** Flow-chart of the study. ABG=arterial blood gas analysis; CPC=cerebral performance category.


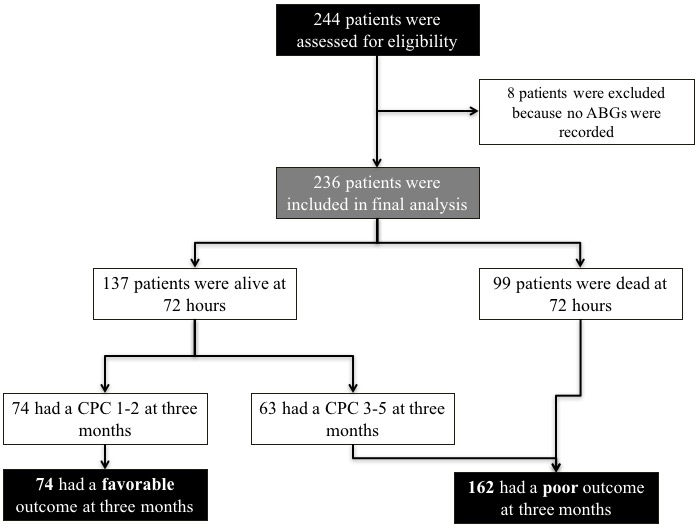


**Figure S2**. Time course of blood lactate concentrations from admission to 48 hours after arrest, according to the occurrence of favourable (CPC 1-2) or poor (CPC 3-5) neurological outcome at 3 months. Data are shown as median [25^th^ – 75^th^ interquartiles].


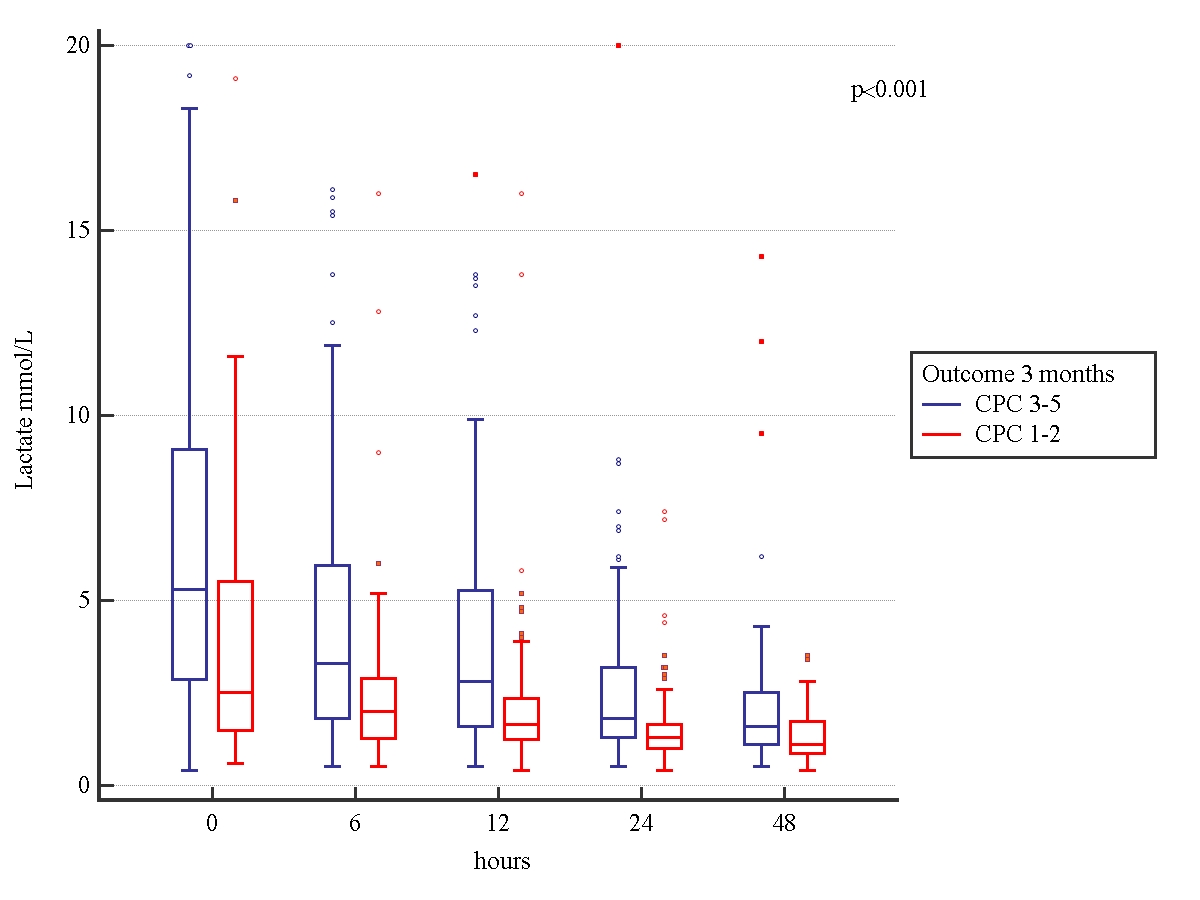


**Figure S3.** Receiving operating characteristics (ROC) curves for the ability of different variables (heart rate [HR], mean arterial pressure [MAP], admission blood lactate concentration and admission cardiovascular SOFA [cSOFA]) to predict unfavourable neurological outcome at three months. The different areas under the curve are: 0.508 (95%CI 0.441-0.576), 0.631 (95%CI 0.564-0.695), 0.689 (95%CI 0.624-0.748) and 0.613 (95% CI 0.546-0.676), respectively.


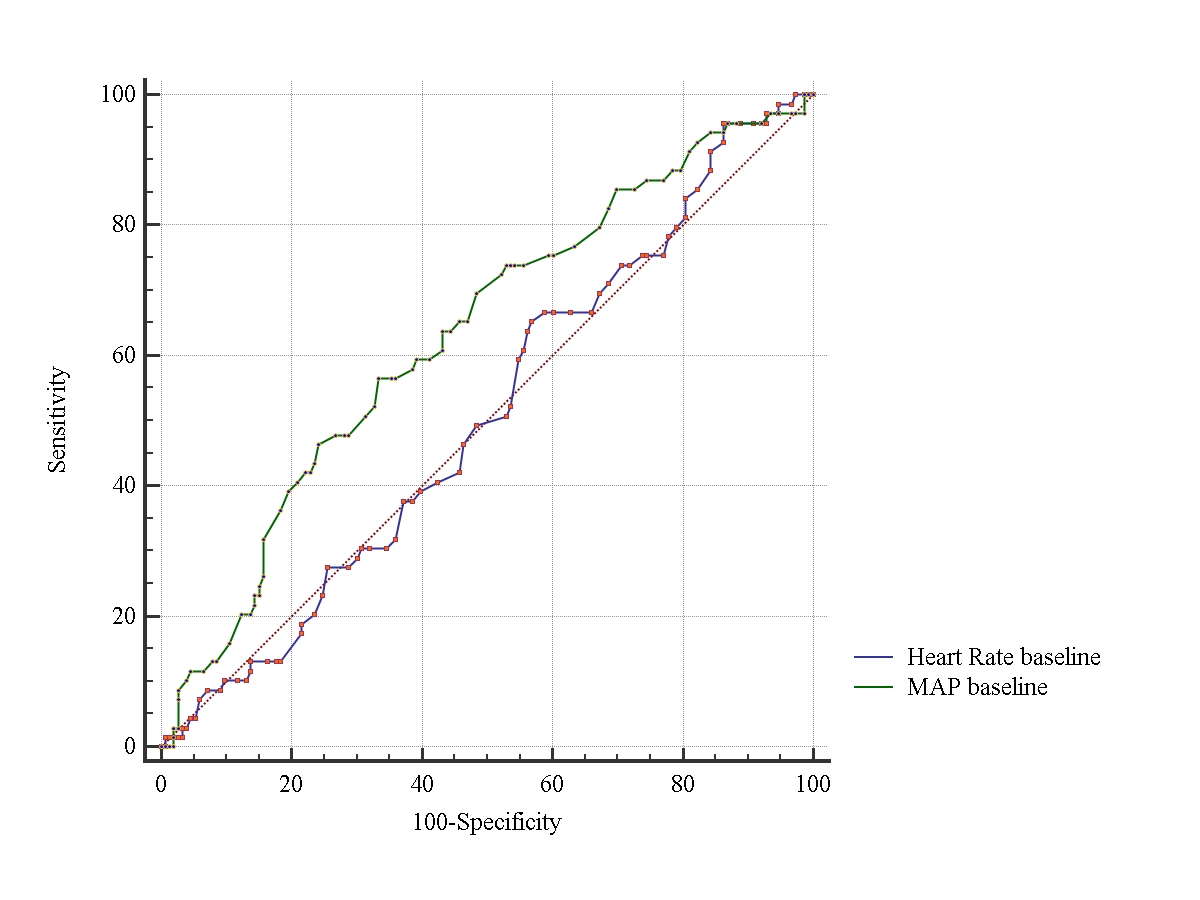


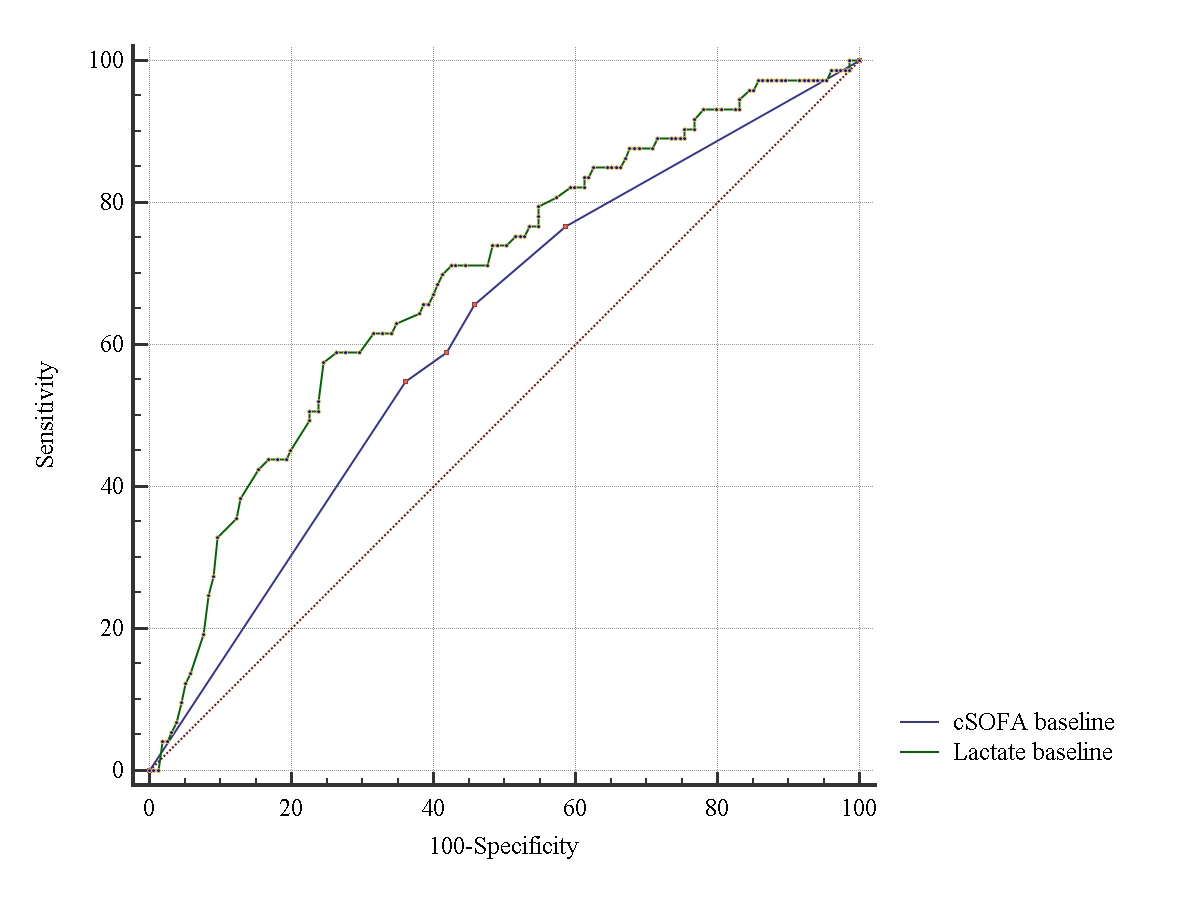


**Table S1.** Arterial blood gas (ABG) results according to the location of the cardiac arrest and neurological outcome

| **Parameter** | **OHCA**  **(137)** | |  | **IHCA**  **(99)** | |  |
| --- | --- | --- | --- | --- | --- | --- |
|  | **CPC 1-2**  **(43)** | **CPC 3-5**  **(94)** | **P value** | **CPC 1-2**  **(31)** | **CPC 3-5**  **(68)** | ***p* value** |
| **ABG** |  |  |  |  |  |  |
| *Lactate admission (mEq/L)* | 2.9 [1.6-6.6] | 4.7 [2.9-78.2] | 0.09 | 1.9 [1.3-3.3] | 5.6 [2.7-11.4] | **<0.001** |
| *Lactate 6h (mEq/L)* | 2.3 [1.5-3.2] | 2.9 [1.9-4.9] | 0.08 | 1.8 [1.1-2.5] | 3.1 [2.0-5.9] | **0.001** |
| *Lactate 12h (mEq/L)* | 1.8 [1.3-2.8] | 2.5 [1.6-4.4] | 0.05 | 1.5 [1.2-2.3] | 2.6 [1.5-3.9] | **0.014** |
| *Lactate 24h (mEq/L)* | 1.3 [1-2.1] | 1.8 [1.3-3.3] | **0.015** | 1.3 [0.9-1.6] | 1.9 [1.3-3.3] | **0.011** |
| *Lactate 48h (mEq/L)* | 1.2 [0.9-1.9] | 1.5 [1.0-2.1] | 0.064 | 1.1 [0.8-1.7] | 1.8 [1.3-2.8] | **0.001** |
| *aDLC _0-6_ (mEq/L*h)* | 0.08 [-0.06 to 0.6] | 0.13 [-0.07 to 0.61] | 0.877 | 0.09 [0-0.2] | 0.4 [0.1-0.7] | **0.045** |
| *aDLC _0-12_ (mEq/L*h)* | 0.1 [0-0.3] | 0.1 [0-0.3] | 0.482 | 0 [0-0.1] | 0.3 [0.1-0.5] | **<0.001** |
| *aDLC _0-24_ (mEq/L*h)* | 0.07 [0.01-0.21] | 0.06 [0-0.2] | 0.532 | 0.03 [0-0.08] | 0.16 [0.03-0.35] | **0.006** |
| *rDLC_0-6_ (%)* | 23 [-27 to 58] | 32 [-22 to 57] | 0.846 | 21 [0-50] | 39 [1-63] | 0.477 |
| *rDLC_0-12_ (%)* | 32 [-16 to 72] | 39 [2-61] | 0.688 | 28 [-5 to 41] | 53 [22-73] | **0.003** |
| *rDLC_0-24_ (%)* | 54 [24-70] | 53 [0-78] | 0.349 | 38 [19-63] | 59 [47-79] | **0.043** |
| *AUC_0-48_, mEq/L*h* | 4.0±2.3 | 4.9±4.6 | 0.117 | 2.8±0.5 | 4.9±0.6 | **0.031** |
| *Peak lactate, mEq/L* | 5.4±3.5 | 6.8±4.1 | 0.06 | 3.9±3.6 | 8.8±5.9 | **<0.001** |
| *pH admission* | 7.25 [7.16-7.35] | 7.25 [7.14-7.34] | 0.606 | 7.33 [7.21-7.41] | 7.28 [7.20-7.42] | 0.404 |
| *PaO_2_ admission (mmHg)* | 141 [72-191] | 127 [83-261] | 0.262 | 143 [86-260] | 105 [70-259] | 0.220 |
| *PaCO_2_ admission (mmHg)* | 45.4±12 | 42.9±16.6 | 0.434 | 40.5±12 | 41.0±13.8 | 0.876 |

CPC=Cerebral Performance Category; CPC 1-2=favourable outcome and CPC 3-5=poor outcome; ABG=arterial blood gas analysis; aDLC=absolute decrease in lactate concentration over time expressed as mEq/L*h; rDLC=relative decrease in lactate concentration over time expressed as %; AUC_0-48_=area under the curve of lactate concentrations for the first 48 hours since admission

**Table S2.** Arterial blood gas (ABG) results according to the initial rhythm.

| **Parameter** | **VF/VT**  **(100)** | **Non-VF/VT**  **(136)** | ***p* value** |
| --- | --- | --- | --- |
| **ABG** |  |  |  |
| *Lactate admission (mEq/L)* | 3.2 [2.3-6.9] | 4.7 [2.4-8.2] | 0.067 |
| *Lactate 6h (mEq/L)* | 2.9 [2.4-4.2] | 2.9 [2.6-5.3] | 0.611 |
| *Lactate 12h (mEq/L)* | 2.1 [1.6-3.3] | 2.4 [1.8-4.0] | 0.226 |
| *Lactate 24h (mEq/L)* | 1.4 [1.1-2.3] | 1.6 [1.1-3.0] | 0.285 |
| *Lactate 48h (mEq/L)* | 1.3 [1.0-1.9] | 1.5 [1.1-2.2] | 0.121 |
| *aDLC _0-6_ (mEq/L*h)* | 0.14 [-0.09 to 0.43] | 0.26 [0-0.69] | 0.088 |
| *aDLC _0-12_ (mEq/L*h)* | 0.11 [0-0.3] | 0.22 [0-0.4] | 0.051 |
| *aDLC _0-24_ (mEq/L*h)* | 0.05 [0.01-0.18] | 0.09 [0.02-0.2] | 0.160 |
| *rDLC_0-6_ (%)* | 26 [-37 to 59] | 32 [0-57] | 0.210 |
| *rDLC_0-12_ (%)* | 30 [-11 to 55] | 44 [11-67] | 0.141 |
| *rDLC_0-24_ (%)* | 48 [10-71] | 55 [24-75] | 0.486 |
| *AUC_-48_, mEq/L*h* | 4.8±4.6 | 4.4±3.4 | 0.543 |
| *Peak Lactate, mEq/L* | 6.9±4.5 | 6.8 ±4.8 | 0.732 |
| *pH admission* | 7.25 [7.11-7.33] | 7.22 [7.11-7.32] | 0.345 |
| *PaO_2_ admission (mmHg)* | 143 [82-242] | 131 [84-241] | 0.588 |
| *PaCO_2_ admission (mmHg)* | 44±16 | 46±18 | 0.673 |

ABG=arterial blood gas analysis; aDLC=absolute decrease in lactate concentration over time expressed as mEq/L*h; rDLC=relative decrease in lactate concentration over time expressed as %; AUC_0-48_= area under the curve of lactate concentrations for the first 48 hours since admission

**Table S3.** Arterial blood gas (ABG) results according to the presence of shock on admission.

| **Parameter** | **Shock**  **(112)** | **No shock**  **(124)** | ***p* value** |
| --- | --- | --- | --- |
|  |  |  |  |
| **ABG** |  |  |  |
| *Lactate admission (mEq/L)* | 7.8 [3.3-10.5] | 2.9 [1.7-4.9] | **<0.001** |
| *Lactate 6h (mEq/L)* | 4.8 [2.6-6.9] | 2.1 [1.5-3.1] | **<0.001** |
| *Lactate 12h (mEq/L)* | 3.6 [1.9-4.9] | 1.8 [1.3-2.6] | **<0.001** |
| *Lactate 24h (mEq/L)* | 1.8 [1.2-3.2] | 1.4 [1.0-2.3] | **0.007** |
| *Lactate 48h (mEq/L)* | 1.6 [1.1-2.5] | 1.2 [0.9-1.8] | **0.004** |
| *aDLC _0-6_ (mEq/L*h)* | 0.32 [0.11-0.64] | 0.08 [-.08 to 0.46] | **0.001** |
| *aDLC _0-12_ (mEq/L*h)* | 0.21 [0.0-0.44] | 0.1 [0 - 0.2] | **0.001** |
| *aDLC _0-24_ (mEq/L*h)* | 0.16 [0.03-0.29] | 0.05 [0-0.12] | **<0.001** |
| *rDLC_0-6_ (%)* | 32 [6-50] | 21 [-30 to 55] | 0.051 |
| *rDLC_0-12_ (%)* | 41 [14 - 62] | 33 [-6 to 61] | **0.035** |
| *rDLC_0-24_ (%)* | 59 [36-78] | 45 [0-68] | **0.002** |
| *AUC_0-48_, mEq/L*h* | 5.4±4.3 | 4.1±3.9 | **0.020** |
| *Peak Lactate, mEq/L* | 9.2±5.6 | 5.1±3.5 | **<0.001** |
| *pH admission* | 7.22 [7.11-7.34] | 7.28 [7.2-7.36] | 0.097 |
| *PaO_2_ admission (mmHg)* | 135 [80-281] | 120 [80.3-199.3] | 0.219 |
| *PaCO_2_ admission (mmHg)* | 42±17 | 43.7±14.8 | 0.182 |

ABG=arterial blood gas analysis; aDLC=absolute decrease in lactate concentration over time expressed as mEq/L*h; rDLC=relative decrease in lactate concentration over time expressed as %; AUC_0-48_= area under the curve of lactate concentrations for the first 48 hours since admissi

**Figure S4**. Panel A: Correlation between decrease in lactate concentrations at 6 hours and blood lactate concentration on admission (r=0.67; p<0.001). Panel B: Correlation between decrease in lactate concentrations at 24 hours and blood lactate concentration on admission (r=0.88; p<0.001).


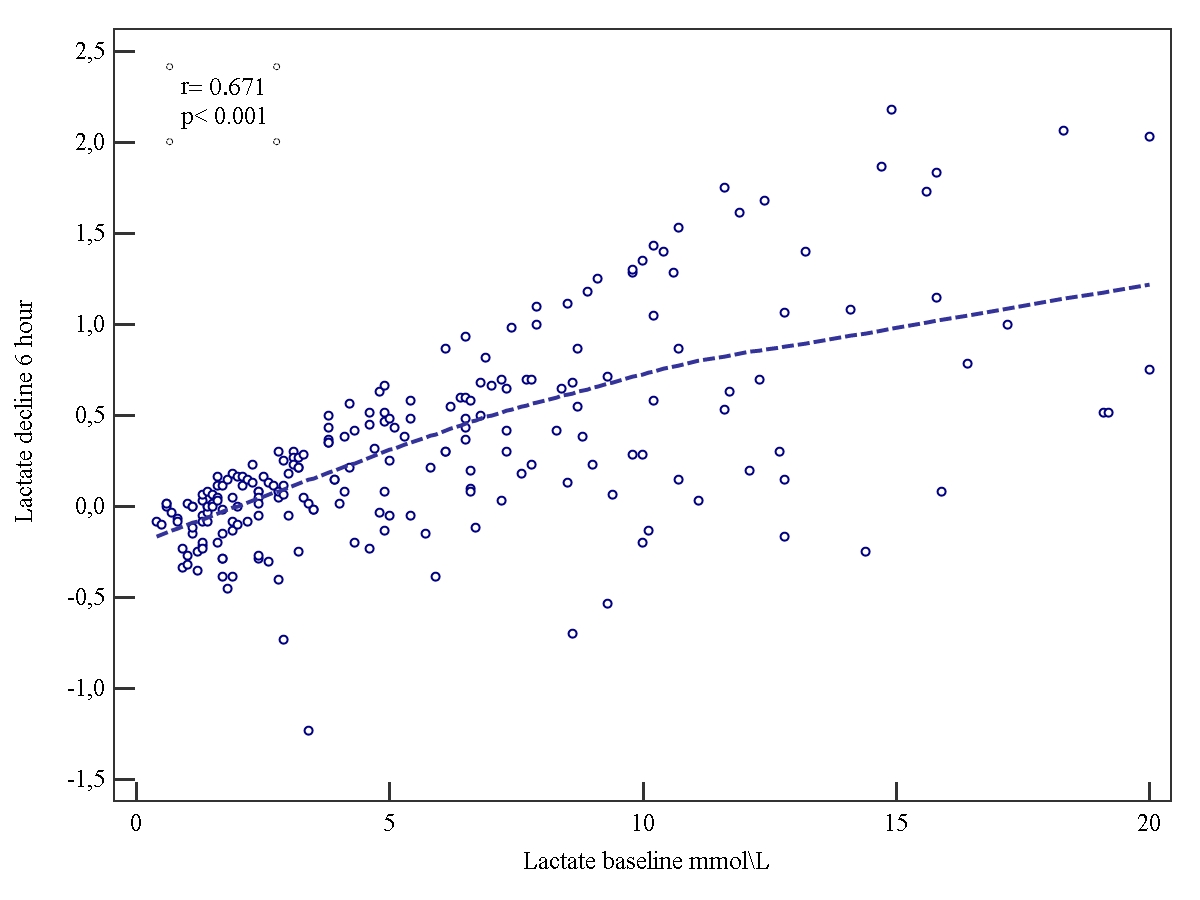


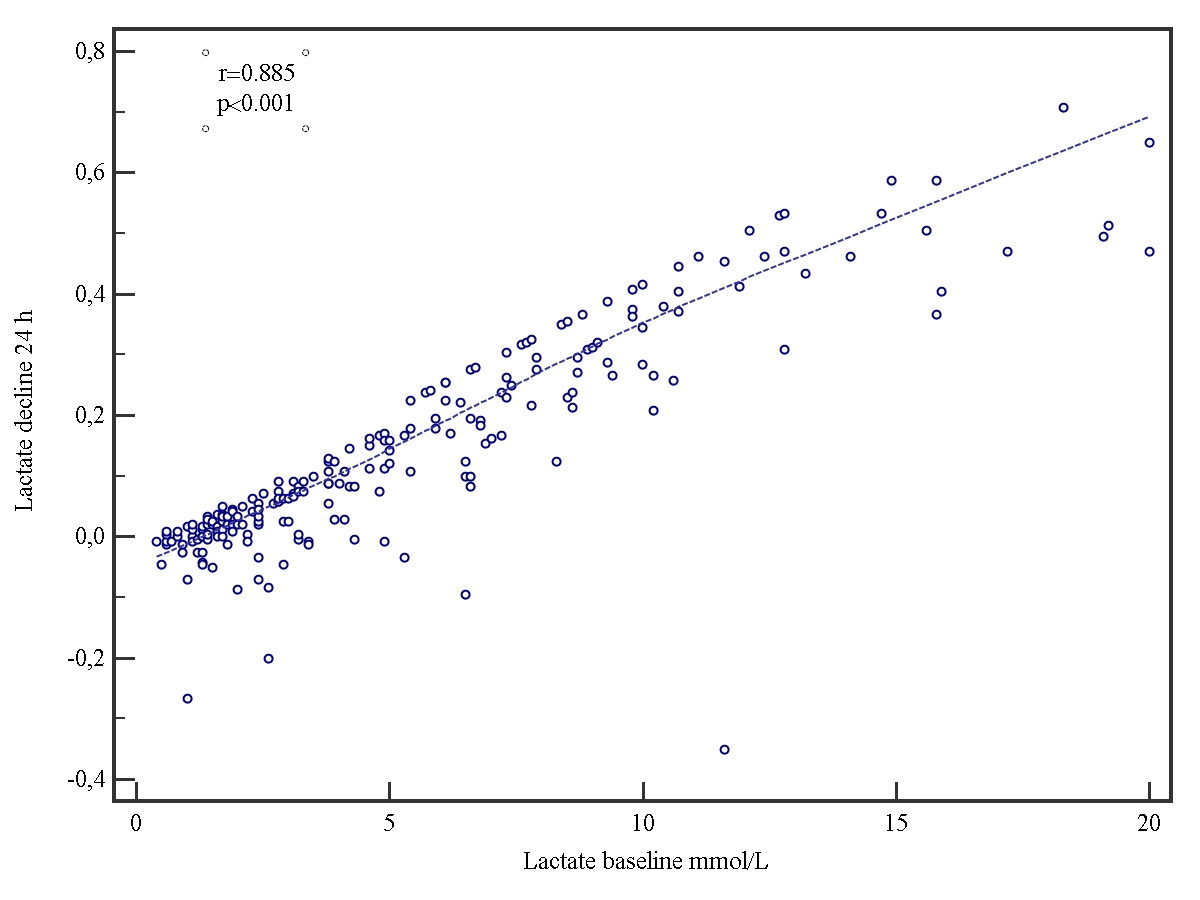

Supplement: Supplementary file 1 — Additional file 1. The additional file contains the supplementary figures and tables referred to in the text. [file 13613_2017_321_MOESM1_ESM.docx]
